# Lung cancer risk in workers occupationally exposed to polycyclic aromatic hydrocarbons with emphasis on the role of DNA repair gene

**DOI:** 10.1007/s00420-022-01926-9

**Published:** 2022-10-26

**Authors:** Gehan Moubarz, Amal Saad-Hussein, Eman M. Shahy, Heba Mahdy-Abdallah, Atef M. F. Mohammed, Inas A. Saleh, Mona A. M. Abo-Zeid, Mahmoud T. Abo-Elfadl

**Affiliations:** 1 Environmental and Occupational Medicine Department, Environment and Climate Change Research Institute, National Research Centre, Giza, Egypt; 2 Air Pollution Research Department, Environment and Climate Change Research Institute, National Research Centre, Giza, Egypt; 3 Genetics and Cytology Department, Genetic Engineering and Biotechnology Research Institute, National Research Centre, Giza, Egypt; 4 Cancer Biology and Genetics Laboratory, Centre of Excellence for Advanced Sciences, National Research Centre, Giza, Egypt; 5 Biochemistry Department, Genetic Engineering and Biotechnology Research Institute, National Research Centre, Giza, Egypt

**Keywords:** Polycyclic aromatic hydrocarbons (PAHs), Alpha 1 antitrypsin (A1AT), APEX1 gene, Tumor biomarkers

## Abstract

**Objective:**

Workers in secondary aluminum production plants are occupationally exposed to polycyclic aromatic hydrocarbons (PAHs). We aimed to monitor the concentrations of PAHs in air and in serum of workers at two secondary aluminum production plants. We also investigated the potential risk of lung cancer development among PAHs exposed workers with emphasis on the role of A1AT mutation and APEX1 gene polymorphisms.

**Methods:**

This study included 177 workers from administrative departments and production lines. Blood samples were obtained for estimation of benzo(a)pyrene diol epoxide albumin adduct (BPDE-Alb adduct), anti-Cyclin-B1 marker (CCNB1) and squamous cell carcinoma antigen (SCCAg). Genes’ polymorphism for human apurinic/apyrimidinic endonuclease (APEX1) and alpha-1-anti-trypsin (A1AT) gene mutation were detected.

**Results:**

There was a significant increase in the level of BPDE-Alb adduct among exposed workers in comparison to non-exposed group. Moreover, 41.67% of exposed workers in El Tebbin had BPDE-Alb adduct level ≥ 15 ng/ml versus 29.6% of workers in Helwan factory. There was a significant increase in tumor markers (SCCAg and CCNB1) among workers whose BPDE-Alb adduct ≥ 15 ng/ml. There was a significant increase in the level of BPDE-Alb adducts in exposed workers carrying homozygous APEX1 genotype *Glu/Glu*. Furthermore, exposed workers with the *Glu/Glu* genotype had high tumor markers levels. There was a significant increase in levels of BPDE-Alb adducts in workers carrying A1AT mutant allele. Moreover, workers with mutant A1AT genotype had significantly high tumor markers (SCCAg and CCNB1) levels.

**Conclusion:**

Therefore, we conclude that aluminum workers may be at a potential risk of lung cancer development due to PAHs exposure. Although PAHs concentrations in air were within the permissible limits, yet evidence of DNA damage was present as expressed by high BPDE-albumin adduct level in exposed workers. Also, elevation of tumor markers (SCCAg and CCNB1) in exposed workers points to the importance of periodic biological monitoring of such workers to protect them from cancer risk.

## Introduction

Burning of fossil fuel has a profound effect on pyrogenic sources in many industries. Polycyclic aromatic hydrocarbons (PAHs) are a group of compounds composed of up to six benzene rings result from incomplete combustion or pyrolysis of organic materials (Luo et al. 2019). They are classified into low molecular weight PAHs (LMW PAHs) that have two or three aromatic rings and high molecular weight PAHs (HMW PAHs), which have four or more aromatic rings. They are emitted either in the gaseous form or in the particulate form (Lee 2010). Workers in industries include coke ovens, iron and steel works, carbon electrode, aluminum works, asphalt manufacture, are exposed to PAHs in huge concentrations.

Several PAHs are known to be highly carcinogenic to humans, such as benza(a)anthracene, chrysene, benzo(b)fluoranthene, benzo(k)fluoranthene, benzo(a)pyrene and benzo(ghi)perylene (Vaezzadeh et al. 2014). Three classes of PAHs exist in the environment; biogenic (minor), petrogenic and pyrogenic (Wang et al 2009).

Workers are mainly exposed to PAHs via inhalation, so workplace air-monitoring of PAHs content is very important because of their toxic, carcinogenic and mutagenic effects on living organisms (HSE 1997; Rusin and Marchwińska-Wyrwał 2014). The carcinogenicity of PAHs is due to their ability to attach DNA forming DNA adduct which generates several disorders that lead to tumor development (Bostrom et al. 2002; Melendez-Colon et al. 1999). Benzo[a]pyrene (BP) is the most important PAHs and is classified as a proven human carcinogen (IARC 2016). It is generally used as an environmental indicator for PAHs (Cherng et al. 1996; Georgiadis and Kyrtopoulos 1999). It is activated to highly toxic metabolite, anti-benzo(a)pyrene diol epoxide albumin adduct (BPDE), which produces a DNA adduct (BPDE-DNA). Most likely, this BPDE-DNA adduct indicates cumulative exposure to BP after metabolite production and intervention of DNA repair enzymes. Therefore, the BPDE-DNA adduct may serve as a useful biomarker for monitoring DNA damage and represents the potential phenotype of DNA damage (Vineis and Perera 2000). BPDE can also bind to human serum albumin and form the BPDE-albumin adduct, which is considered a useful marker for monitoring PAHs exposure (Chung et al. 2010).

Occupational exposure to PAHs is associated with lung cancer (De Matteis et al. 2012; IARC 2018). Squamous cell carcinoma (SCC) of the lung is a type of non-small cell lung cancer. It often occurs in the central part of the lung or in the main airway. This cellular transformation is also associated with tobacco smoking, which contains more than 40 potential carcinogenic agents including PAHs (Ettinger rt al. 2013; Sabbula and Anjum 2021). Several tumor markers have been investigated so far, in which the most commonly used is squamous cell carcinoma antigen (SCCAg) (Henkenberens et al. 2016). Additionally, researches in non-small cell lung cancer have shown that high levels of cyclin B1 (CCNB1) are associated with non-small cell lung cancer. The studies also suggested that the expression CCNB1 may be used as a predictive marker in patients with early stage non-small cell lung cancer (Soria et al. 2000; Wang et al. 2020).

Genetic factors are responding to occupational and environmental hazards (Chiu et al. 2013; McHale et al. 2012; Patel et al. 2012). The use of genetic information could possibly be more powerful in assessment and reduction of health problems related to occupational hazards, and in improving methods for protection of workers at high risks (NRC 2010; EC and IPPC 2014). So genes can be used as biomarkers of susceptibility to exposures. It may be a major cause of the variability in health response to occupational hazards among similarly exposed individuals (Schwartz et al. 2011).

DNA repair mechanisms are needed to prevent DNA damage resulting from exposure to high levels of PAHs, but individuals differ in their DNA repair capacity, and this may be a risk factor for development of cancer in individuals with low DNA repair capacity (Benhamou and Sarasin 2000). Polymorphisms in several DNA repair genes involved in nucleotide base excision repair pathways (BER) have been studied, but their functional activity remains to be clearly described. The human apurinic/apyrimidinic endonuclease (APEX1) protein is the main enzyme in the repair of apurinic/apyrimidinic sites in DNA strand breaks (Li et al. 2015). Therefore, genetic polymorphisms of APEX1 genes may determine individual’s susceptibility to DNA damage which may be attributed to PAHs exposure. The resulting genetic mutations can serve as an early diagnostic biomarkers for diseases related to environmental and occupational exposures (Cortessis et al. 2012). Alpha-1 antitrypsin (A1AT) is a protease inhibitor sensitized primarily in the liver. It inhibits the neutrophil elastase activity in the lung and hence can protect it from proteolytic damage (Dahl et al. 2005). A1AT mutation has been associated with increased susceptibility to develop lung diseases. The most common allele is associated with normal function and is labeled M. While, the mutant alleles are labeled S and Z alleles. Individuals of the A1AT S and Z alleles are over-presented in patients with lung cancer (Yang et al. 1999).

The present work was done to monitor PAHs concentrations in airborne suspended particulate matter (SPM) and in serum of workers by estimation of benzo(a)pyrene diol epoxide albumin adduct (BPDE-Alb adduct); as a potential exposure biomarker for carcinogenic high molecular weight PAHs in the secondary aluminum production industry. Moreover, it also investigates lung cancer risk among PAHs exposed workers with emphasis on the role of A1AT mutation and APEX1 gene polymorphisms on their susceptibility to lung cancer development.

## Methodology

### Study design

This study was a cross-sectional comparative study, conducted in two major aluminum factories in Helwan and El-Tebbin areas, Cairo, Egypt. In the two factories, aluminum is manufactured through secondary process by recycling aluminum scrap into aluminum sheets that can be used again. During the secondary process, the scrap is collected, isolated, and placed into a melting furnace to be molten at temperatures ranging from 1300 to 1400 ℉. Secondary aluminum is then used in many of the applications where primary aluminum is utilized.

### Subjects

This study was conducted on 177 workers from the two selected factories: 30 workers from the administrative departments (non-exposed) and 147 from production line in secondary aluminum smelter (exposed) of the two included factories. The exposed workers were classified into two groups: 87 in group 1 and 60 in group2 from Helwan and El-Tebbin factories respectively. Their working duration was more than 5 years with working 8 h/day. Group 3 workers comprised the non-exposed workers were from the administrative departments of the two factories; they have never been occupationally exposed to PAHs. The workers in the three groups were matched in their age, duration of exposure in years and smoking habits.

Workers who reported recent chest complaints or had chronic respiratory diseases not related to their working exposures were excluded from the study. After obtaining a written informed consent from all the included workers, they filled a detailed personal questionnaire including age, socioeconomic status and smoking habit. Environmental and occupational present and past histories were registered (duration of exposure in years, shift duration in hours, and type of exposure).

### Workplace monitoring

- El-Tebbin industrial company
- This company works in aluminum industry and copper rolling. It was established in industrial area El-Tebbin. Its aluminum production is about 6000 tons annually, where aluminum raw materials are melted in casting ovens and poured into cold rolling mill, and then the product is transferred to Annealing ovens area. After that, part of the product is transferred to the manufacture of household utensils. Another part is transferred to Gravity area for melting and poured into different casting molds. The third part is formed to be used in refrigerators then transferred to heat treatment furnaces. The surface treatment is finished by electrochemical method or electrostatic coating. After that, the final inspection is carried out, and then packaged for internal delivery or for export.
- Helwan factory

- The factory works in aluminum production. It was established on an area of more than 50000 square meters in industrial Area–Helwan. The production capacity of the factory is 12000 tons annually where aluminum raw materials are melted in smelting furnaces and poured in cylinders of various sizes. The products (as sectors) are transferred to the exit table, then cooled by air coolers and transferred to the storage table to be cut to the required lengths. The products are transferred to the stage inspection for thickness and surface and then transferred to the heat treatment furnaces. The surface treatment of the sectors is finished by electrochemical method or by electrostatic painting in all colors. After that, the final inspection is carried out, and then packaged to be delivered internally or for export.

### Monitoring of PAHs

At each aluminum factory, samples of suspended particulate matter (SPM) were collected from the air at different working areas one day a week during the year 2020. The samples were collected on pre-weighed glass fiber filters (Whatman GFA type) using low volume sampler technique and vacuum pump was calibrated at a rate of 9 L per min for a period of 8 h (the shift duration of the workers) (Mukhopadhyay et al.^2020^). The filters were reweighed after sampling and the difference in weight was SPM. SPM samples were stored in unused standard plastic bags and transferred to the laboratory. All samples were air-dried at room temperature in dark place (Hassan 2006).

PAHs in SPM samples were Soxhlet extracted with a solvent solution (the mixture of n-hexane and dichloromethane, 500 ml/L of each) for 24 h according to Chiu et al. (2013a). The extracts were then concentrated to about 3 ml on a rotary evaporator for the clean-up procedure (Drotikova et al. 2020). The collected eluent from the clean-up procedure was concentrated to 1 ml on a rotary evaporator and stored at 4 °C till analysis (Hassan 2006). A Gas Chromatograph (GC) (Hewlett-Packard HP6890), fitted with a Flame Ionization Detector (HD) was used. HP-5 (30 m × 320 μm × 0.25 μm) capillary column with hydrogen as carrier gas was used. The GC was calibrated with a diluted standard solution of 16 compounds of PAH mixture, Supelco, Inc., Bellefonte, PA, and the concentrations of the target PAH compounds were quantified using this external standard solution.

The concentrations of the following PAHs were determined: naphthalene (NA), Acenaphthylene (ACY), acenaphthene (ACE), phenanthrene (PHE), fluorene (FLU), anthracene (ANT), fluoranthene (FLT), pyrene (PYR), benzo(a)anthracene (BaA), chrysene (CRY), benzo(b)fluoranthene (BbF), benzo(k)fluoranthene (BkF), benzo(a)pyrene (BaP), dibenzo(a,h)anthracene (DBA), indeno (1,2,3-c,d)pyrene (IND), and benzo(ghi)perylene (BGP).

PAH sources in a matrix can be confirmed by calculated the total index as the sum of single indexes. When the total index is > 4, we consider PAHs originating prevalently from high temperature processes (combustion) while index values lower than four indicate prevalently low temperature sources (petroleum products) (Orecchio 2010).

$${\text{Total index}} = {\text{Fl}}/\left( {\text{Fl + Py}} \right)/0.4 + {\text{An/}}\left( {\text{An + Ph}} \right){/0}{\text{.2}} + {\text{B[a]A/}}\left( {\text{B[a]A + Chr}} \right){/0}{\text{.1 + IND/}}\left( {\text{IND + B[ghi]P}} \right){/0}{\text{.5}}$$

where Fl is Fluoranthene, Py is Pyrene, An is Anthracene, Ph is Phenanthrene, B[a]A is Benzo[a]pyrene, Chr is Chrysene, IND is Indeno(1,2,3-c,d)pyrene, and B[ghi]P is Benzo[ghi]perylene. In addition, 0.4, 0.2, 0.1, 0.5 are the most predominant ratio factors for Fl/(Fl + Py), An/(An + Ph), B[a]A/(B[a]A + Chr), IND/(IND + B[ghi]P respectively found in most previous studies.

Toxicity equivalent (TEQ) was calculated for PAHs using the following equation

$${\text{TEQ}}\left( {\text{ng/g}} \right){\text{ = B[a]A}} \times {0}{\text{.10 + Chry}} \times {0}{\text{.01 + B[a]P}} \times {1}{\text{.00 + B[ghi]Per}} \times {0}{\text{.01}}$$

where B[a]A is benzo[a]anthracene, Chry is Chrysene, B[a]P is Benzo[a]pyrene, and B[ghi]Per is Benzo[ghi]perylene. In addition 0.10, 0.01, 1.00, 0.01 are toxicity factors used for B[a]A, Chry, B[a]P, B[ghi]Per respectively in most previous studies.

### Biological monitoring

About 10 ml of blood samples was collected from the included workers. The blood samples were divided into two aliquots. The first aliquot was centrifuged for separation of serum to determine all serological tests. The second aliquot was collected in a vacationer containing EDTA as anticoagulant for RNA and DNA extraction and stored at − 80 °C until analysis.

The following analyses were preformed:

1. Estimation of benzo(a)pyrene diol epoxide albumin adduct (BPDE-Alb adduct) in serum by sandwich enzyme-linked immunosorbent assay (ELISA) according to manufacturer’s instruction (SinoGeneClon Biotech Co. Ltd. China, Catalog No: SG-15696).
2. Estimation of anti-Cyclin-B1 marker (CCNB1) and squamous cell carcinoma antigen (SCCAg) by Enzyme-linked immunosorbent assay (ELISA) according to the manufacturer’s instructions (SinoGeneClon Biotech Co. Ltd. China).
3. Detection of genes polymorphism for human apurinic/apyrimidinic endonuclease (APEX1) rs1130409 (Asp148Glu) and alpha-1-anti-trypsin (A1AT) gene mutation using polymerase chain reaction–restriction fragment-length polymorphism (PCR–RFLP) method as follows:
4. Step1: Genomic DNA was isolated from blood samples collected from workers using the QIAamp DNA Blood Mini kit following the manufacturer's protocol and quantified using spectrophotometer and stored at − 20 °C.
5. Step2: The polymorphisms and mutation were analyzed using the polymerase chain reaction–restriction fragment-length polymorphism (PCR–RFLP) method. PCR was performed in a total volume of 25 μL containing 100 ng of genomic DNA, 1 × PCR Master Mix (Tiangen), and 5 pmol of each primer. Using primers specific and suitable conditions to gene (Table 1, 2).

**Table 1:** PCR primers and specific condition for A1AT, and APEX1 rs1130409 gene

Gene	Primers (reverse and forwards)	*T* (°C)	References
*A1AT* S & Z mutation	S (forward) 5′-TGA GGG GAA ACT ACA GCA CCT CG-3′, S (reverse) 5′-AGG TGT GGG CAG CTT CTT GGT CA-3′, Z (forward)5′-ATA AGG CTG TGC TGA CCA TCG TC-3′, Z(reverse) 5′-TTG GGT GGG ATT CAC CAC TTT TC-3′	Initial denaturation for 5 min. at 94 °C; 35 cycles of 1 min. at 94 °C, 1 min. at 55 °C, and 2 min. at 72 °C; final extension for 10 min. at 72 °C	Tazelaar et al. (1992)
*APEX1* gene rs1130409 (Asp148Glu)	F 5′-CTG TTT CAT TTC TAT AGG CTA-3′, R 5′-AGG AAC TTG CGA AAG GCT TC-3′	Initial denaturation at 95 °C–5 min, 35 cycles of 95 °C–20 s, 55 °C–20 s, 72 °C–20 s and final extension at 72 °C–10 min	Lunn et al. (2000)

**Table 2:** Methodology for restriction-RFLP product length

Gene	Restriction enzyme	RFLP products (bp)
*A1AT* Sand Z mutation	Taq1	Fragments of (157 + 22 bp) and (100 + 21 bp) were detected for the wild type M allele, a 179 bp fragment for the Z allele and a 121 bp fragment for the S allele
*APEX1* gene rs1130409	FspBI	*Asp/Asp*: 164 bp; *Asp/Glu*: 164 bp, 144 bp, 20 bp; *Glu/Glu*: 144 bp, 20 bp

- Total mRNA was isolated using the RNeasy Mini Kit protocol according to the manufacturer’s instructions (Cat. No. 74104_Qiagen, Hilden, Germany). Single-stranded cDNA was prepared from 1 μg of total RNA and 0.5 μg of oligo-dT primer using the reverse transcription-PCR Kit (Cat. No. 205310_Qiagen, Germany). The cDNA was then amplified by PCR at 42 °C for 15 min. Quantification of cDNA by RT-PCR using individual target gene specific primers: apurinic/apyrimidinic endonuclease (*APEX1*) (NM_080649.2;catalog number: PPH02201A-Agilent) and alpha-1-antitrypsin (*A1AT*) (NM_000295.4;catalog number: PPH06905E-Agilent) with SYBR Green I-based RT-PCR cycler and ROXTM dye as a condition for fluorescence normalization (reference dye) according to the protocol of Quanti NovaTM SYBR^®^ Green PCR Kit (Agilent, CA, USA). The target mRNA gene expression level was standardized by the house keeping gene GAPDH (NM_002046.5; catalog number: PPH00150F-Agilent) and calculated according to delta-delta CT method.
- Human apurinic/apyrimidinic endonuclease (*APEX1*) level and alpha-1-antitrypsin (*A1AT*) level were detected using a commercial enzyme-linked immunosorbent assay (ELISA) kit according to the manufacturer’s instructions (SinoGeneClon Biotech Co. Ltd. China).

### Statistical analysis

The results were computerized; quantitative data were presented as mean ± SD, while qualitative data were presented as number and percent. Statistical analysis was done using Minitab version 20. Comparisons between two groups were done using independent *t* test, for comparisons between more than two groups one-way analysis of variance (ANOVA) was used. Skewness data were analyzed using non-parametric methods. Matrix plot Pearson Correlation was used to study the relationships between quantitative dependent variables. Level of significance was set at the *P* < 0.05.

## Results

### Air monitoring results

**Fig. 1:**
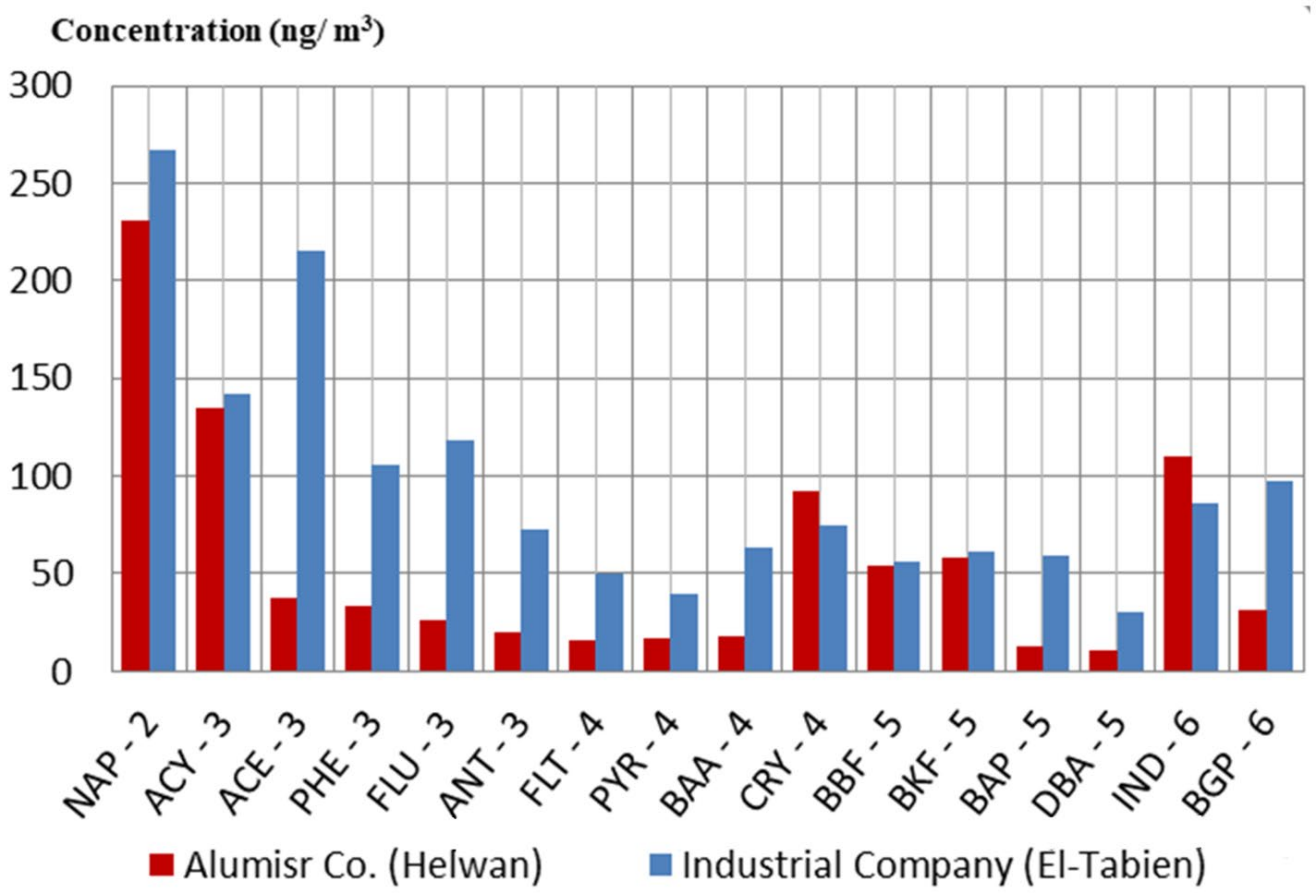
The average concentrations of individual PAHs compounds (ng/m^3^) at sampling sites

The average concentrations of gas chromatograph results of PAHs individual compounds of the two factories (El-Tebbin and Helwan) are shown in Fig. 1 and Table 3. The concentrations of most common PAHs individual compounds were higher in EL-Tebbin compared to Helwan factory.

**Table 3:** Showed the chromatography of standard mixtures and samples.

PAHs		Area under curve (mAu)													Retention time (min.)
		Standard	Sample												
			Cold rolling mill area	Annealing ovens area	Gravity area	Evaporators workshop	Casting ovens area	Administration offices	Smelting furnaces area	Press ovens area	The oxidation ponds area	The painting workshop	Export warehouse office	Administration offices	
Naphthalene	NAP-2	1,134,602.27	293,499.3	534,989.0	1,605,123.7	257,745.3	446,717.3	1,028,016.5	1,028,016.5	398,078.6	2,706,212.7	1,352,718.4	4,316,839.8	19,818.6	5.61
Acenaphthylene	ACY-3	2,598,053.35	312,491.2	4,780,732.0	4,510,241.4	1,764,824.7	2,729,135.3	1,270,485.4	1,270,485.4	363,505.2	1,661,413.7	1,734,250.7	4,733,578.0	16,355.2	5.86
Acenaphthene	ACE-3	2,763,026.44	753,828.2	3,694,459.5	4,085,238.7	1,630,740.8	2,151,097.5	869,389.5	869,389.5	430,648.6	1,092,835.7	3,603,007.1	1,507,167.0	13,058.6	6.03
Phenanthrene	PHE-3	3,022,753.94	291,489.8	5,545,721.2	3,855,471.0	1,901,697.3	2,611,071.8	1,896,117.3	1,896,117.3	495,232.2	1,482,195.9	2,157,675.0	1,835,340.8	19,582.2	6.19
Fluorine	FLU-3	4,137,207.07	614,616.0	2,196,837.1	1,857,740.5	1,875,192.1	3,779,661.1	1,110,493.6	1,110,493.6	815,439.8	2,041,225.3	2,281,414.6	1,674,834.8	22,589.8	7.01
Anthracene	ANT-3	5,401,687.53	254,750.4	4,417,819.3	4,669,781.7	3,550,273.2	4,465,815.4	2,245,568.6	2,245,568.6	605,318.0	1,841,374.2	3,552,924.2	1,088,394.7	20,588.0	13.12
Fluoranthene	FLT-4	6,463,779.35	336,773.5	1,744,122.0	2,014,438.9	2,362,585.0	3,028,009.3	1,278,582.4	1,278,582.4	920,111.6	1,391,086.9	3,020,913.1	1,230,585.4	22,001.6	14.45
Pyrene	PYR-4	6,825,708.59	148,175.8	2,187,543.2	4,249,777.9	2,431,727.3	4,291,361.7	2,095,920.3	2,095,920.3	804,690.7	1,656,630.7	2,160,374.7	1,511,379.8	20,900.7	24.12
Benzo(a)anthracene	BAA-4	7,618,383.27	447,142.3	2,929,288.6	1,592,876.0	7,199,334.3	4,334,332.0	6,227,687.5	6,227,687.5	991,278.9	2,911,233.8	2,524,314.6	1,136,868.1	29,708.9	28.39
Chrysene	CRY-4	4,517,819.96	132,193.3	1,926,679.6	3,194,169.0	1,695,686.9	1,621,573.5	1,065,421.3	1,065,421.3	494,898.0	2,310,703.2	1,309,471.5	1,675,037.4	19,908.0	29.98
Benzo(b)fluoranthene	BBF-5	9,218,691.18	538,398.1	2,041,663.2	2,502,378.2	2,172,998.1	7,893,805.0	1,510,851.0	1,510,851.0	972,421.5	1,691,430.5	2,569,756.3	5,717,671.1	47,201.5	31.79
Benzo(k)fluoranthene	BKF-5	6,600,043.63	462,721.2	2,598,023.2	3,083,281.1	1,759,762.5	2,299,203.7	1,355,096.6	1,355,096.6	711,130.8	2,182,813.8	4,323,566.8	2,418,674.2	42,300.8	33.91
Benzo(a)pyrene	BAP-5	8,127,969.7	570,381.8	2,141,464.2	4,101,118.5	1,672,100.9	2,795,439.9	1,046,933.0	1,046,933.0	506,821.4	1,116,298.7	3,536,869.1	1,565,316.7	12,201.4	34.75
Dibenzo(a,h)anthracene	DBA-5	9,823,999.21	244,973.1	1,853,847.7	3,279,989.3	3,187,016.1	2,812,302.6	1,807,104.6	1,807,104.6	646,870.0	1,139,038.9	2,999,465.6	1,289,277.9	14,700.0	35.83
Indeno(1,2,3-c,d)pyrene	IND-6	2,852,810.16	115,453.4	3,333,180.8	2,461,528.0	2,906,701.2	3,905,754.3	891,675.7	891,675.7	342,195.0	4,227,255.2	1,294,439.4	1,357,243.7	24,905.0	39.13
Benzo(ghi)perylene	BGP-6	2,837,819.23	228,595.1	1,803,710.0	4,091,306.1	1,861,489.0	2,542,517.1	4,698,183.3	4,698,183.3	386,736.0	1,247,928.0	2,684,512.3	1,199,468.0	18,306.0	43.71

**Fig. 2:**
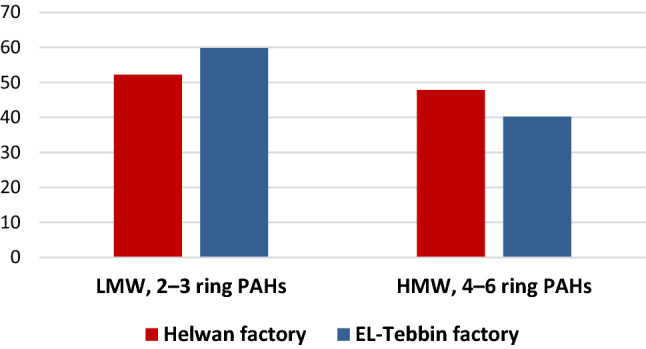
Relative distribution of PAHs according to molecular weight

All 16 PAHs listed by EPA were detected in all particulate samples. Low molecular weight PAHs (LMW, 2–3 ring PAHs) were the most predominant PAHs with contribution 52.2% and 59.8% at the industrial companies Helwan and EL-Tebbin, respectively. While high molecular weight PAHs (HMW, 4–6 ring PAHs) were the less predominant PAHs in samples with lower contribution 47.8 and 40.2% at industrial company Helwan and EL-Tebbin, respectively (Fig. 2). These results suggested that environmental PAHs pollutions were basically attributed to combustion activities. Where, LMW-PAHs were originated from low-temperature combustion processes. While, HMW-PAHs were originated from high-temperature combustion processes. These results indicate that the anthropogenic sources (release of un-combusted petroleum products and combustion processes) were the responsible for PAHs concentrations in the working environment of the sampling sites.

Thus, the air samples containing high molecular weight PAHs were more frequent in Helwan factory (47.8%) than in EL-Tebbin factory (40.2%) (Fig. 2).

**Fig. 3:**
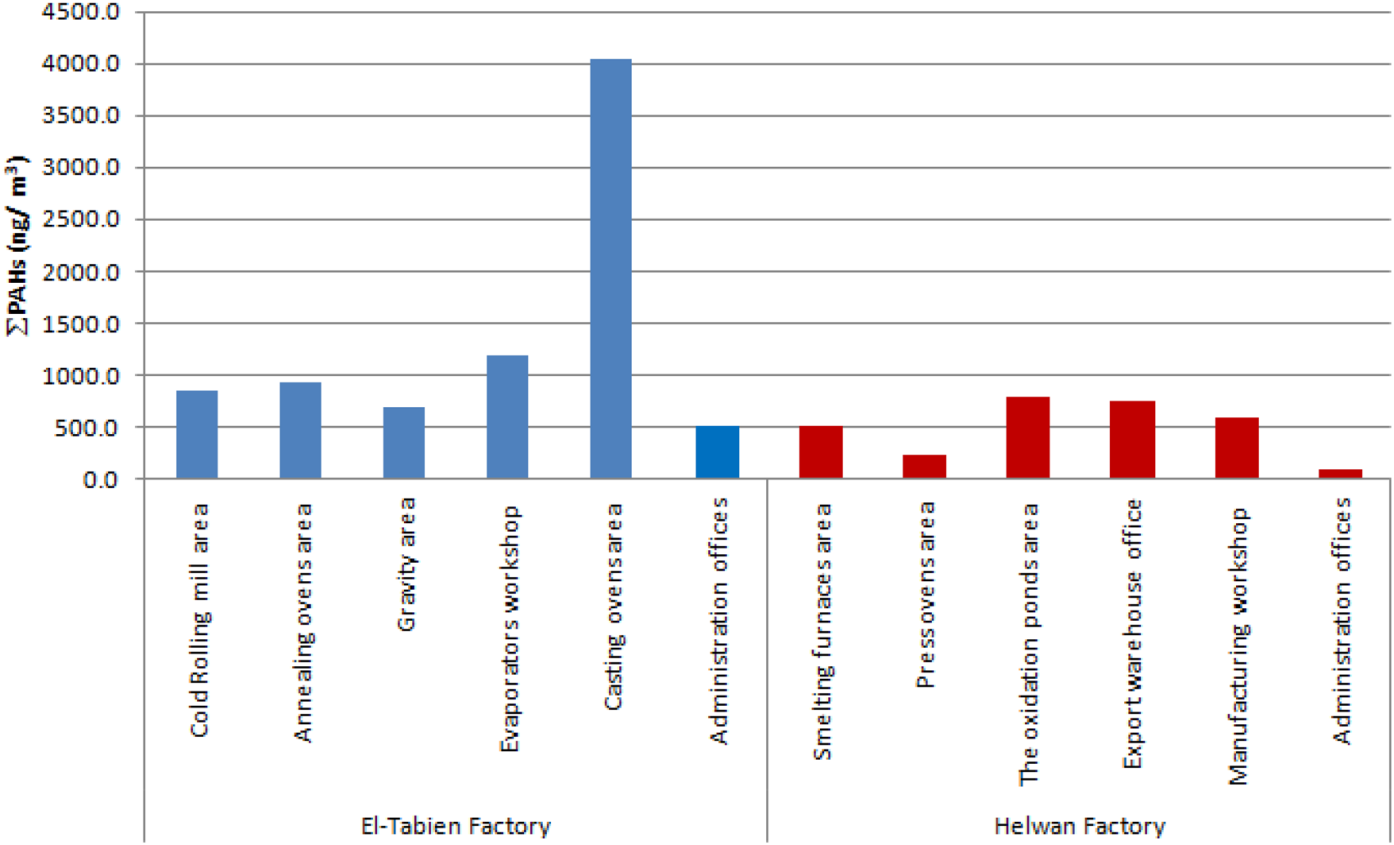
PAHs compounds at different partitions in sampling sites

Figure 3 shows ∑PAHs (ng/m^3^) at different partitions in the sampling sites. It was found that the highest concentration in EL-Tebbin factory was 4041 ng/m^3^, which was detected at the area of the casting ovens area. While the highest concentration in Helwan factory was 786 ng/m^3^ and 752 ng/m^3^, which was detected at the oxidation ponds area and at export warehouse office, respectively.

Table 4 showed the total index and Toxicity equivalent (TEQ) at the sampling sites. The total indexes ranged between (6.1–12.3) and (6.5–11.2) at EL-Tebbin and Helwan, respectively. The results showed that the total indexes are > 4, which confirmed that the PAHs compounds in SPM samples were originating prevalently from high temperature processes (combustion). In addition, the toxicity equivalents (TEQ) of PAHs are higher at casting ovens area in EL-Tebbin factory and at smelting furnaces area in Helwan factory.

**Table 4:** Toxicity equivalent (TEQ) and total index

Sampling sites	Toxicity equivalent (TEQ) (ng/m^3^)	Total index (ng/m^3^)
EL-Tebbin factory		
Cold rolling mill area	22.3	9.1
Annealing ovens area	39.1	6.1
Gravity area	22.2	12.3
Evaporators workshop	31.1	6.9
Casting ovens area	220.4	11.5
Helwan factory		
Smelting furnaces area	16.4	11.2
Press ovens area	5.7	8.1
The oxidation ponds area	14.8	6.5
Export warehouse office	15.4	6.8
Manufacturing workshop	6.1	9.1

### Biological monitoring results

Table 5 showed a significant increase in the serum levels of BPDE-Alb adduct (high molecular weight PAHs, five rings) among exposed workers compared to non-exposed group. While, no significant difference was detected in the smokers compared to non-smokers exposed workers (*P* = 0.6). Also significant increase was detected in the levels of BPDE-Alb adduct among exposed workers from Helwan factory in comparison to that in the exposed workers from El-Tebbin Factory.

**Table 5:** Comparison of BPDE-Alb adduct levels in exposed and non-exposed groups

Variables	BPDE-Alb adduct (ng/ml) (mean ± SD)	*P* value
Total exposed workers (147)	19.5 ± 18.5	0.01
Non-exposed group (30)	10.52 ± 3.6	
Exposed smoking workers (61)	20.27 ± 18.3	0.6
Exposed non-smoking workers (86)	18.5 ± 16.8	
Exposed group 1 (87)	21.6 ± 19.1	0.04
Exposed group 2 (60)	16.5 ± 12.3	

The exposed workers included in this study were then divided into two subgroups (according to median of BPDE-Alb adduct levels), into workers with BPDE-Alb levels ≥ 15 ng/ml and workers with BPDE-Alb adduct levels < 15 ng/ml.

**Fig. 4:**
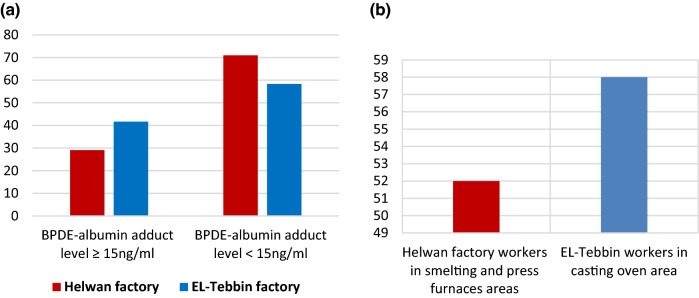
**a** Percentage of workers with different BPDE-Alb adduct levels in studied two factories, **b** the workplace of the workers with BPDE-Alb adduct levels ≥ 15 ng/ml in the two factories

About 29.6% of workers from Helwan factory had BPDE-Alb adduct ≥ 15 ng/ml, while, 41.67% of exposed workers from EL-Tebbin factory had BPDE-Alb adduct ≥ 15 ng/ml, without significant differences (*Chi square* = 2.837, *P* value = 0.092), (Fig. 4a). About 58% of EL-Tebbin workers were with BPDE-Alb adduct ≥ 15 ng/ml were from casting oven area, and about 52% of Helwan factory workers with BPDE-Alb adduct ≥ 15 ng/ml were from smelting and press furnaces areas (Fig. 4b).

Table 6 showed significant increase of the tumor biomarkers (SCCAg and CCNB1) in the workers with BPDE-Alb adduct ≥ 15 ng/ml compared to those with BPDE-Alb adduct < 15 ng/ml.

**Table 6:** Comparison of tumor biomarkers between the exposed workers with BPDE-Alb adduct ≥ 15 ng/ml and those with BPDE-Alb adduct < 15 ng/ml

	Workers with BPDE-Alb adduct ≥ 15 ng/ml		Workers with BPDE-Alb adduct < 15 ng/ml		Independent *t* test	
	Mean	SD	Mean	SD	*t* test	*P *value
SCCAg (pg/ml)	548	56	242.1	14	5.25	0.001
CCNB1 (ng/ml)	14.4	1.8	6.88	0.39	4.19	0.001

**Fig. 5:**
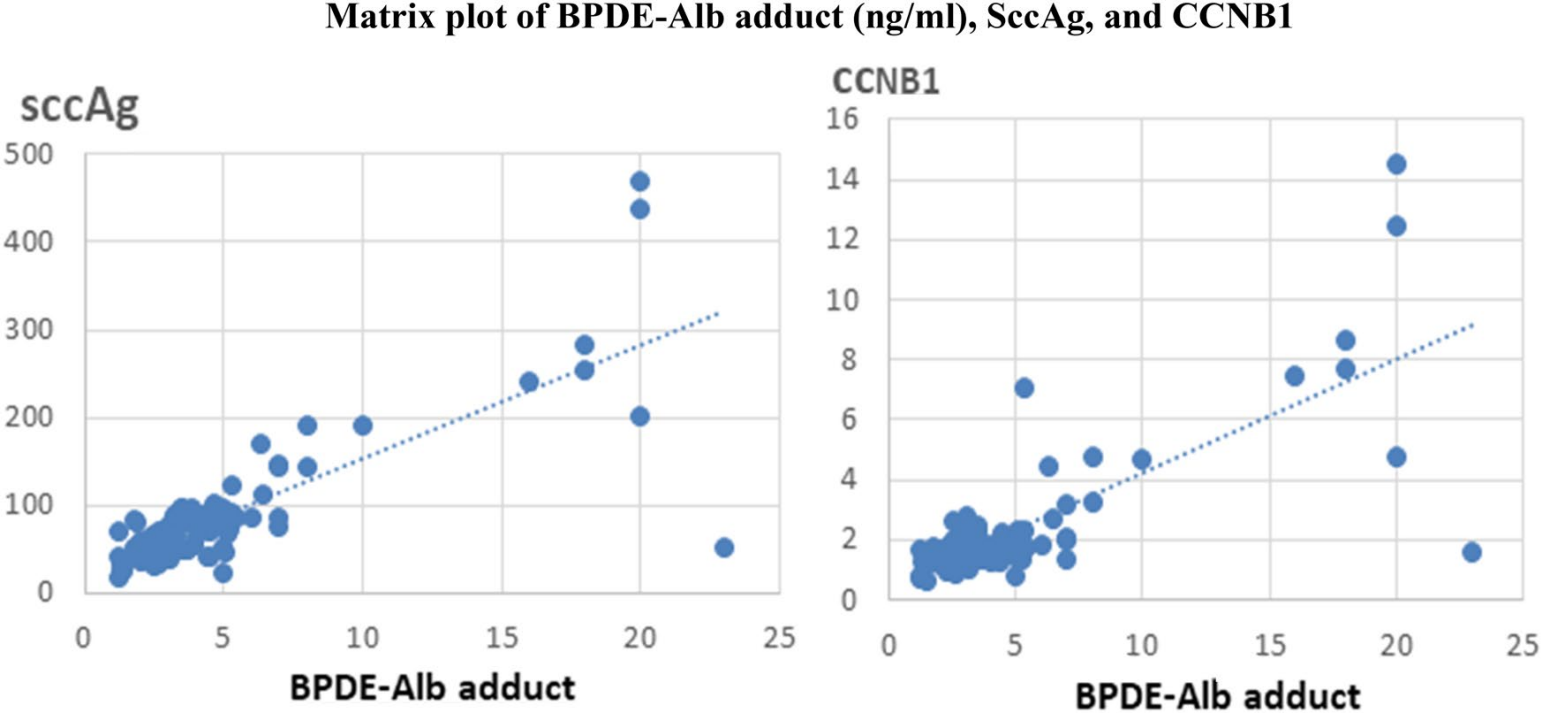
Relationship between the BPDE-Alb adduct levels and tumor biomarkers in the exposed workers (*n* = 147)

Figure 5 showed a significant positive correlations between BPDE-Alb adduct levels and the tumor biomarkers among exposed workers (SCCAg *r* = 0.8, *P* < 0.0001, and CCNB1 *r* = 0.77, *P* < 0.0001).

It was shown in Table 7 that, there was a significant increase in levels of BPDE-Alb adduct levels in the exposed workers carrying homozygous *Glu* genotype compared to workers with homozygous *Asp* and heterogeneous *Asp/Glu* genotypes. Moreover, the exposed workers with the APEX1 *Glu/Glu* genotype had high tumor biomarkers levels than *Asp/Asp* and *Asp/Glu* genotypes.

**Table 7:** Comparison of the levels of BPDE-Alb adduct and the tumor biomarkers according to the different APEX1 gene polymorphisms among exposed workers

Variables	*Asp/Asp*	*Asp/Glu*	*Glu/Glu*	*P* value
	Mean ± SD	Mean ± SD	Mean ± SD	
BPDE-Alb adduct (ng/ml)	22.36 ± 3.62	20.1 ± 3.7	28.38 ± 4.35^(a,b)^	0.05
SCCAg (pg/ml)	372.41 ± 33.4	309.52 ± 62.5	523.7 ± 74.38^(a,b)^	0.01
CCNB1 (ng/ml)	9.15 ± 0.71	7.83 ± 1.19	14.85 ± 2.35^(a,b)^	0.01

NB: ^a^significant elevation compared to *Asp/Asp*

^b^significant elevation than *Asp/Glu*

**Fig. 6:**
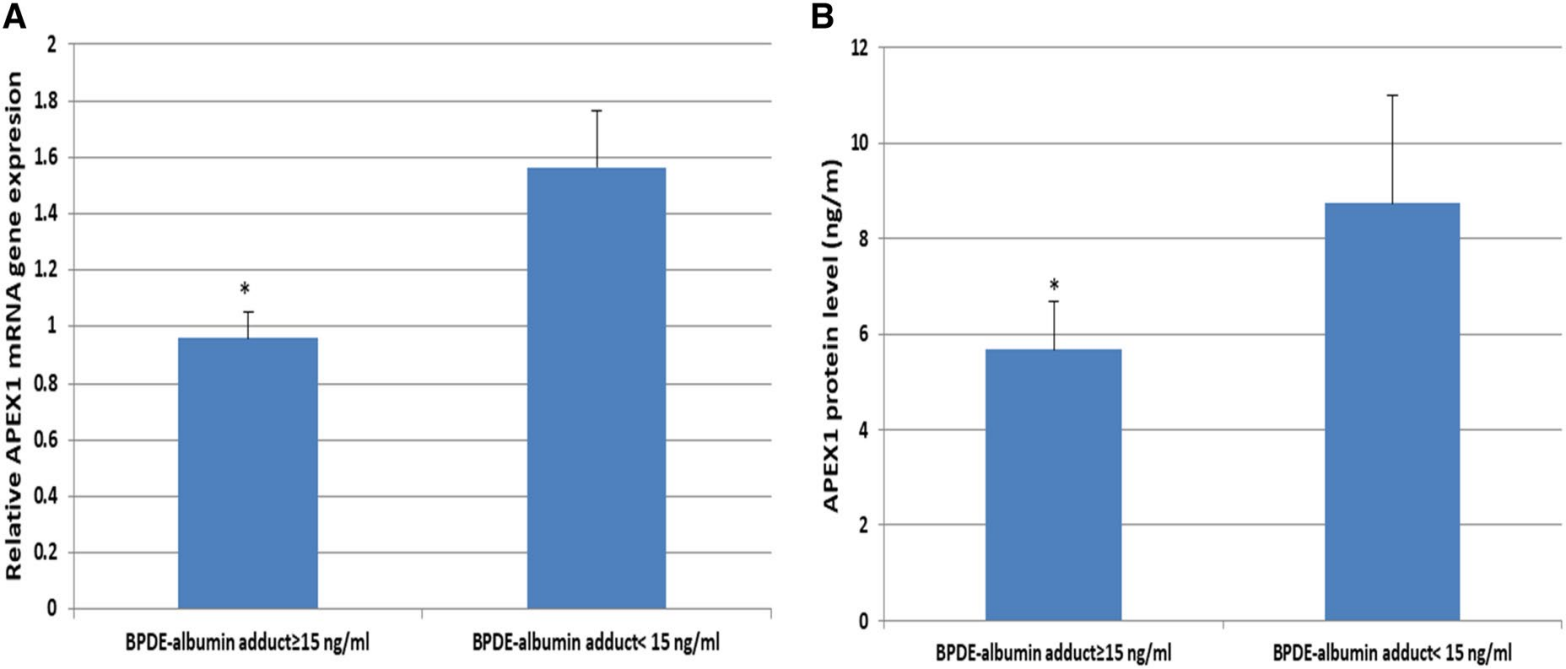
**A** Relative expression APEX1 gene among workers with different BPDE-Alb adduct levels, **B** Levels of APEX1 protein among workers with different BPDE-Alb adduct levels. *p < 0.05

APEX1 gene expression was significantly downregulated in BPDE-Alb adduct ≥ 15 ng/ml workers compared to BPDE-Alb adduct < 15 ng/ml workers (1.58 fold, *p* < 0.0001) (Fig. 6A). Additionally, a statistically significant decrease in APEX1 protein levels was observed in the BPDE-Alb adduct ≥ 15 ng/ml group as compared with those with BPDE-Alb adduct < 15 ng/ml group (Fig. 6B).

**Fig. 7:**
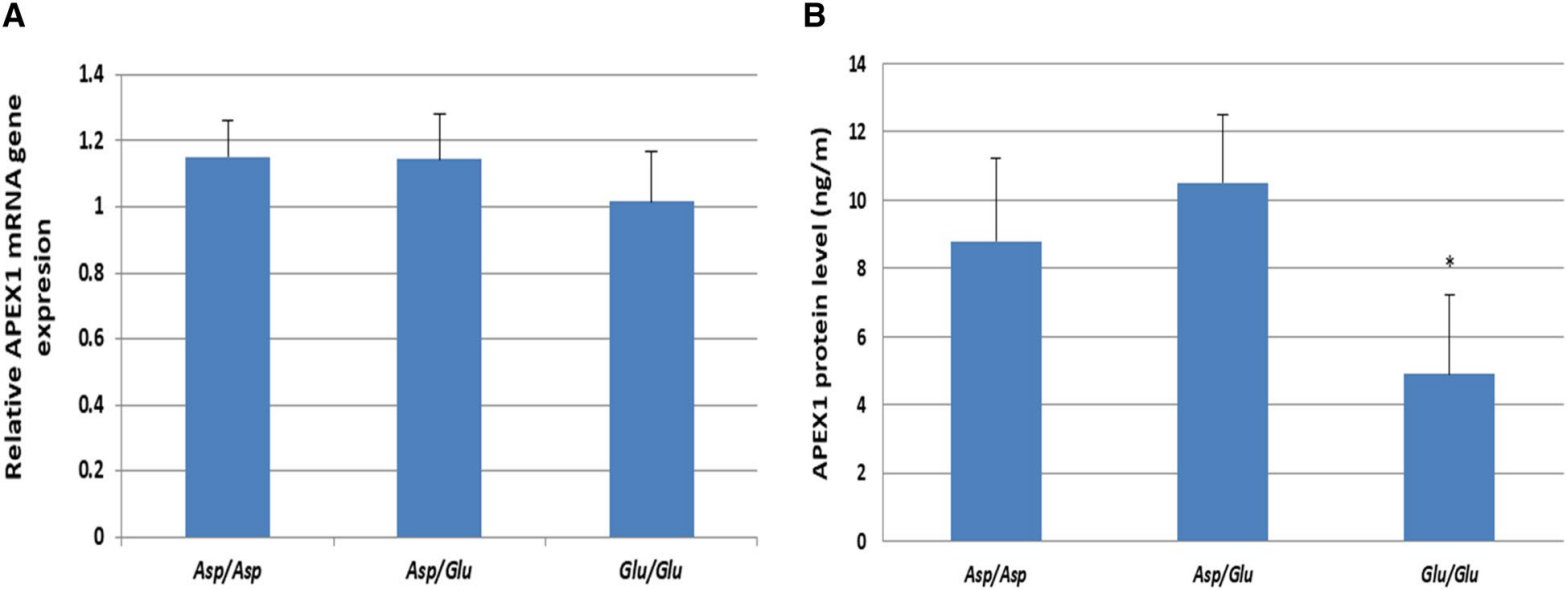
**A** Relative expression APEX1 gene among workers with different APEX1 genotypes. **B** Levels of APEX1 protein among workers with different APEX1 genotypes. *p < 0.05

The expression level of APEX1 gene was also related to the three different genotypes. A non-significant difference in mRNA expression of APEX1 gene was observed in the three different genotypes (Fig. 7A). While, a significant decrease APEX1 protein level was observed in homozygote *Glu/Glu* genotype workers compared to *Asp/Asp* and *Asp/Glu* genotypes workers (Fig. 7B).

Using PCR/RFLP analysis of S (264Glu3Val) and Z (342Glu3Lys) mutations, Table 8 showed that there were significant increases in the levels of BPDE-Alb adduct and the tumor biomarkers (SCCAg and CCNB1) in workers carrying A1AT mutant allele compared to workers with the wild type allele.

**Table 8:** Comparison of the levels of BPDE-Alb adduct, and tumor biomarkers among exposed workers

Variables	MM (wild type)	S and Z mutation	*P* value
	Mean ± SE	Mean ± SE	
BPDE-Alb adduct (ng/ml)	18.35 ± 1.75	44.59 ± 16.41	0.01
SCCAg (pg/ml)	408.11 ± 35.4	890.8 ± 293.7	0.01
CCNB1 (ng/ml)	9.9 ± 0.7	29.2 ± 10.3	0.04

**Fig. 8:**
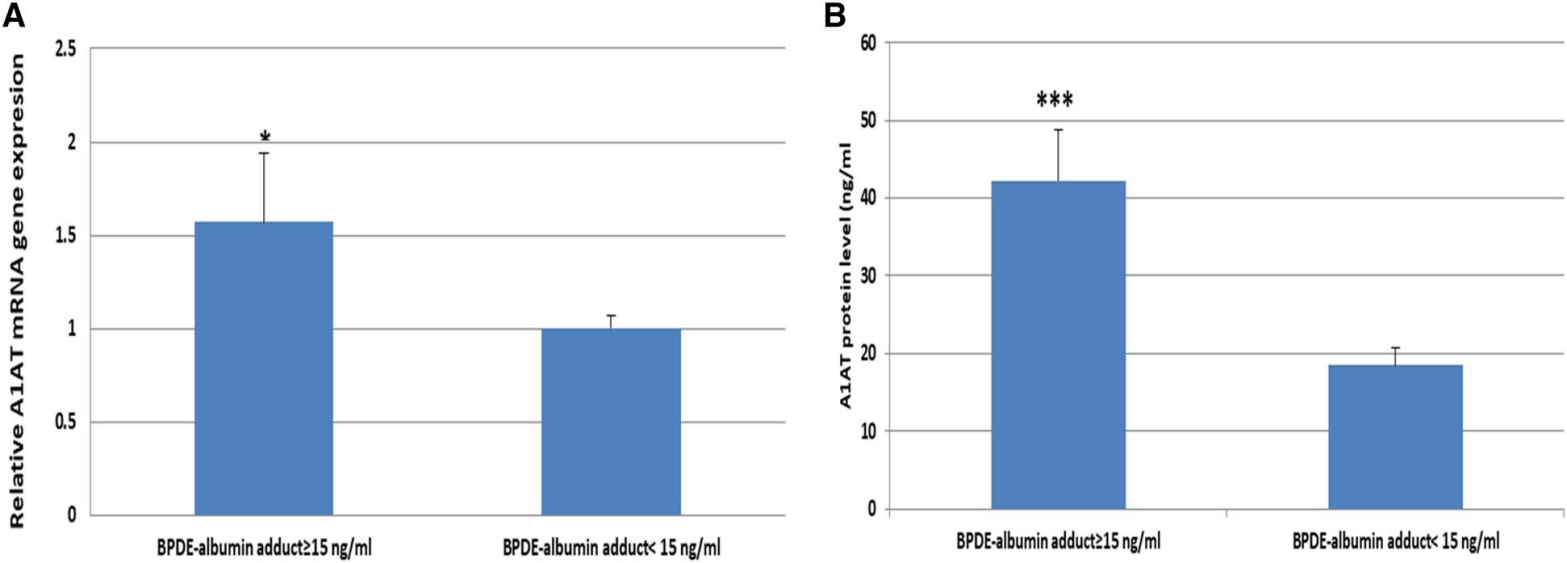
**A** Relative expression A1ATgene among workers with different BPDE-albumin adduct levels. **B** Levels of A1AT protein among workers with different BPDE-alb adduct levels. *p < 0.05, ***p < 0.0001

The relative expression of A1AT in exposed workers showed a significant up-regulation expression in BPDE-Alb adduct ≥ 15 ng/ml workers compared to BPDE-Alb adduct < 15 ng/ml workers (Fig. 8A). Additionally, statistical significant increase in A1AT protein level was observed in workers with BPDE-Alb adduct ≥ 15 ng/ml compared to those with BPDE-Alb adduct < 15 ng/ml (Fig. 8B).

**Fig. 9:**
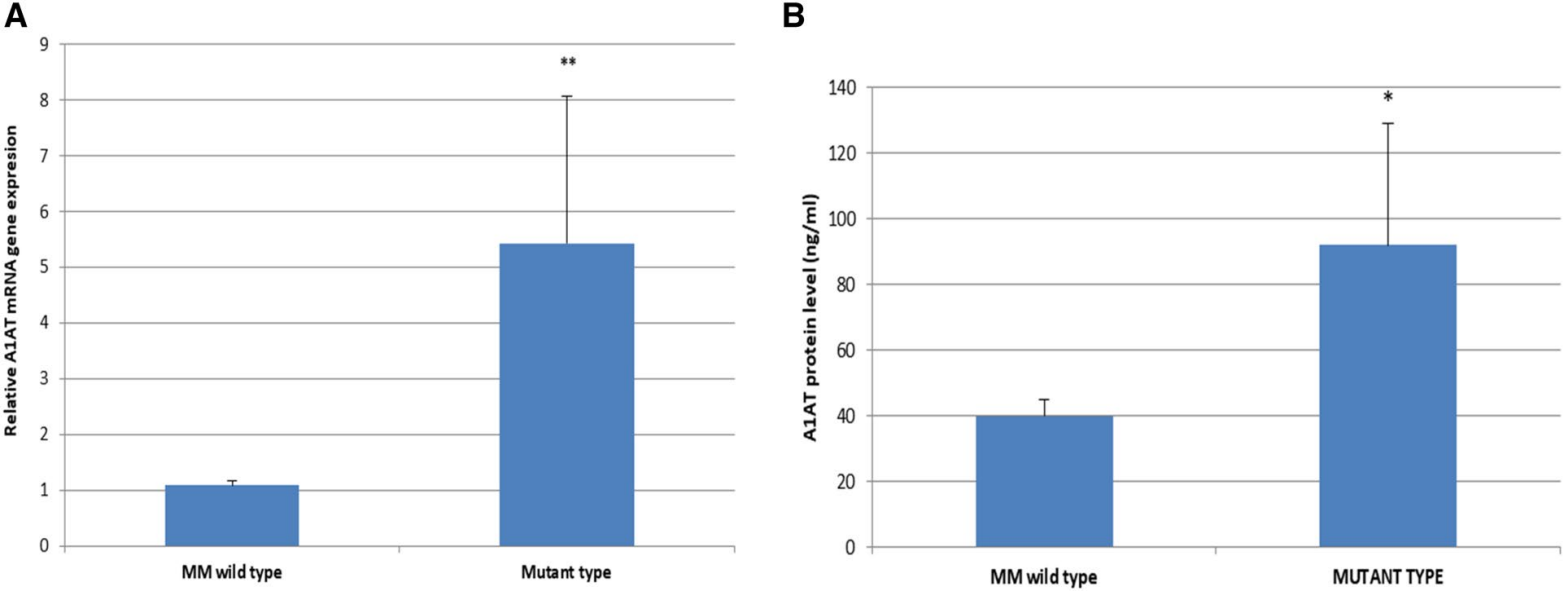
**A** Relative expression of A1AT gene among workers with different A1AT genotypes. **B** Levels of A1AT protein among workers with different A1AT genotypes. *p < 0.01, **p < 0.001

In addition, the S and Z mutant type workers showed a significant up-regulation in A1AT gene expression compared to MM wild type workers (Fig. 9A). Similarly, a significant increase of A1AT protein levels was detected in the mutant A1AT workers compared to wild type (Fig. 9B).

**Fig. 10:**
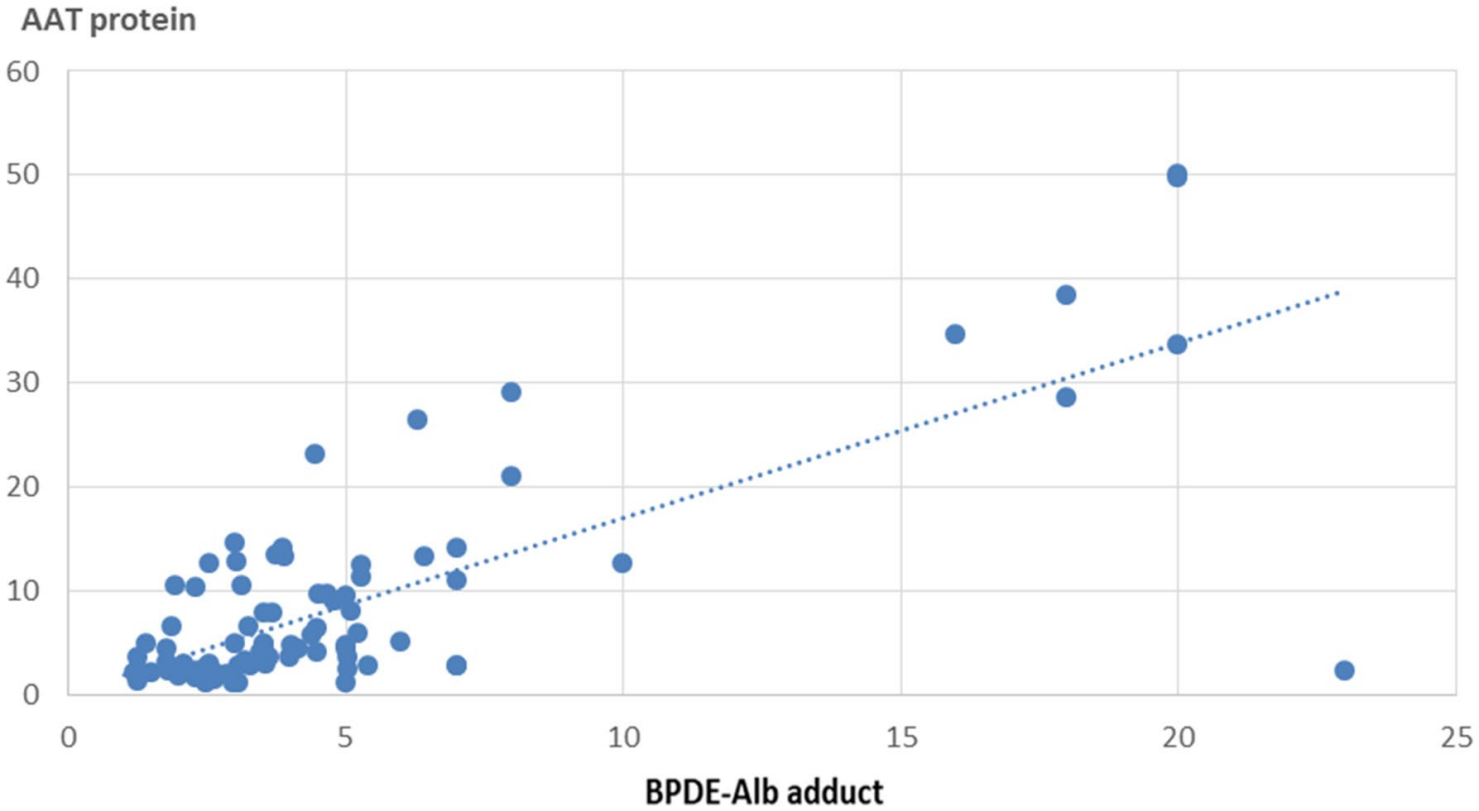
Relationship between the BPDE-Alb adduct level, A1AT protein level among exposed workers (*n* = 147)

Figure 10 showed significant positive correlations between BPDE-albumin adducts levels and A1AT protein levels among exposed workers (*P* < 0.0001).

## Discussion

Workers in aluminum production are exposed to PAHs compounds. Occupational exposures to PAHs emissions in this industry are considered to be potentially carcinogenic. In this study, we observed that the levels of most individual PAHs were higher in EL-Tebbin factory than in Helwan factory, where the work areas in EL-Tebbin are located in an industrial area affected by high concentrations of pollutants. Recently, Zhu et al. (2021) proved that building location, designs and ventilation influence pollutant concentrations. It is clear that the design of the working area plays a major role in pollutant concentrations and hence health impacts on workers.

The results of the current study showed that the individual PAHs concentrations were attributed to combustion activities, due to the predominance of low molecular weight PAHs (with 2–3 ring PAHs) in the two factories which arise from un-combusted petroleum products and combustion processes. These results were in agreement with Nitsche et al. (2017) who found that LMW-PAHs originated from low-temperature combustion processes, while HMW-PAHs result from high-temperature combustion processes. In addition, Patel et al. (2020) stated that incomplete combustion is the main source of PAH emissions by various industrial activities such as aluminum production. Similarly, our results indicate that the anthropogenic sources (release from incomplete combustion of petroleum products and combustion processes) were responsible for the concentration of PAHs in the sample area.

The results in the present study indicated that casting ovens are responsible for most of PAHs emissions in EL-Tebbin factory (4041 ng/m^3^); the oxidation ponds area (786 ng/m^3^) and export warehouse office (752 ng/m^3^) are responsible for most of PAHs emissions in Helwan factory. The total indexes of PAHs ranged from (6.1–12.3 ng/m^3^) to (6.5–11.2 ng/m^3^) at EL-Tebbin and Helwan factories, respectively. The higher total index more than 4 ng/m3 implies that PAHs also originated from high temperature combustion processes, which according to those found by Orecchio (2010). The toxicity equivalent (TEQ) of PAHs was also higher in the casting ovens area, which may be attributed to the higher concentrations of PAHs in these partitions.

Although air measurement of PAHs dust in the current work was within the acceptable levels (OSHA permissible exposure limit 0.2 mg/m^3^ = 200,000 ng/m^3^), there was possibility of increased risk of cancer development among exposed workers according to OSHA (2021). Hence, long-term exposure to low levels of some PAHs has caused cancer in laboratory animals.

Benzo(a)pyrene (BP) is the most common high molecular weight PAH causing human and animal cancer (IARC 2010; Rengarajan et al. 2015). In the present study, the serum concentrations of BPDE-albumin adduct (active metabolite of BP) was measured as a potential exposure biomarker for PAHs. Detection of BDE-albumin adduct levels could be performed using either Eliza (Chung et al. 2013) or other chemical specific assays (Käfferlein et al.^2010^). A significant increase in the average levels of BPDE-albumin adduct (ng/ml) was detected in the present study among the exposed workers in comparison to the non-exposed workers. Several studies observed that the elevated level of BDPE-albumin adduct among workers was correlated with high molecular weight PAHs in different industries (Zhang et al. 2012; Alhamdow et al. 2018).

Regarding to smoking habits, the current study showed no significant changes in the levels of BPDE-Alb adduct among smokers and non-smoker workers. This indicates that the elevation in BPDE-Alb adduct levels among workers was mostly related to occupational exposure to the high levels of PAHs detected in their working environment. The same results were mentioned previously, tobacco smoke is considered to be one of the important sources of PAHs, but, occupationally exposure levels to PAHs in aluminum and coal production were more higher (Hecht 2003; Pavanello et al. 2006).

Regarding to work partitions, about 58% of EL-Tebbin workers with BPDE-Alb adduct level ≥ 15 ng/ml were from casting oven area. As a higher concentration (4041 ng/m3) was detected in the air samples collected from the casting ovens area in EL-Tebbin. While about 52% of Helwan factory workers with BPDE-Alb adduct level ≥ 15 ng/ml were from smelting and press furnaces areas. Obviously, PAHs are always produced in complex mixtures during the combustion process of coal and oil (Kim et al. 2013).

Several studies have shown an association between exposure to PAHs and risk of lung cancer development (Rota et al. 2014; Ifegwu et al. 2015). Cyclin B1 (CCNB1) was reported to be overexpressed in many cancers such as lung cancer. Also, squamous cell carcinoma antigens (SCCAg) is expressed in normal epithelial tissues, and elevated in several benign lesions of squamous cells, but, has been proven to be important in the diagnosis of lung cancer (Oh 2016; Taniguchi 2009). Accordingly, to that these two biomarkers were used in the present study to examine the risk of PAHs carcinogenicity among the exposed workers, as PAHs may enhance these serum tumor biomarkers which may further promote tumor initiation. This is attributed to the ability of PAHs to damage DNA by generating BPDE metabolite, which can damage DNA by forming DNA adducts (BPDE-DNA) through covalent binding to the amino group of guanine or adenine (Wei et al. 2000).

Chu et al. (2011) found that levels of SCCAg, and CCNB1 were elevated in early-staged lung cancer patients. Thus, use of SCCAg and CCNB1 biomarkers for distinguishing early-staged cancers from benign masses could be of great value. Currently, these two tumor biomarkers were used for prediction and early diagnosis of cancers, and the significant positive correlations detected in the present work between BPDE-albumin adduct levels and the two tumor biomarkers among exposed workers pointed to increase risk of the possibility to develop cancers among them. Thus, the included aluminum workers are at risk of developing cancers as a result of their exposure to high concentrations of the carcinogenic PAHs. Therefore, it was recommended that further efforts, modifications and safe PAHs guidelines are still needed for better protection from occupational exposure to PAHs that may reduce the incidence of cancer (Petit et al. 2019).

Exposure to PAHs is an important cause to cancers, and polymorphisms altering DNA repair capacity may lead to synergistic effects with PAH-induced genotoxic effects and cancer risks (De Marini et al. 2001). One of the most important explanations for results in human biomonitoring studies is the inter-individual variations in DNA repair capacities that may aid the effects of the exposures. Recognizing differences between individuals helps to provide information on making decisions to limit the risks of exposure in sensitive workers. The human protein APEX1 is an enzyme that limits the rate in the DNA base excision repair (BER) pathway. BER plays a key role in eliminating the adduct effects of toxic PAHs (Lu et al. 2001; Chou et al. 2002).

APEX1 is known to have a multifunction, as it facilitates the BER pathway in repairing damaged DNA, and it also acts as a co-activator for several transcription factors, which are involved in cancer promotion and progression (Izumi et al. 2000; Ando et al. 2008). In the present study, significant relationships were detected between APEX1 genotypes and the levels of BPDE-albumin adduct and the tumor biomarkers. The increase levels of BPDE-albumin adduct and tumor biomarkers were significantly detected in the workers carrying *Glu/Glu* allele compared to the other two genotypes. This could mean the exposed workers with Glu*/Glu* allele were supposed to be at high cancer risks than the other exposed workers.

These results generally agree with a previous study that revealed higher levels of plasma BPDE-Alb adduct in workers carrying *Glu* genotypes (Li et al. 2015). In addition, many studies have evaluated the association between *Glu* variants and cancers risk, particularly lung cancers (Ito et al. 2004; Agaçhan et al. 2005; Jin et al. 2014). In contrast, another study reported a protective role of APEX1 *Glu* allele against lung cancer risk (Deng et al. 2011). So, on exposure to genotoxic agents such as PAH, the homozygous APEX1 *Glu* carriers may be more susceptible than *Asp* carriers to develop DNA damage as a result of the exposures.

Little is known about the mechanism or influence of extracellular secretion of APEX1 in the conditions of PAHs exposure. Here, we showed significant down-regulation of APEX1 mRNA; as confirmed by RT-qPCR, and significant decrease in the protein expression levels; measured by ELISA, among the workers with high BPDE-Alb adduct level (≥ 15 ng/ml) when compared to those with low BPDE-Alb adduct level (< 15 ng/ml). These findings suggested reduction in DNA repair ability as a result of exposure to high concentration of PAHs resulting in accumulation of albumin adducts that may lead to DNA damages and mutations, which may eventually become malignant. Recently, Zhou et al (2022) showed that the expression of some metabolic and DNA repair genes was induced by BP and inhibited by FO or omega-3 fatty acids in liver, but not lung.

Regarding to the different APEX1 genotypes, the current study observed non-significant differences in mRNA expression of APEX1 gene. Similarly, Mollica et al. (2016), reported that APEX1 gene expression didn`t attribute to wild-type levels, and suggests additional mechanisms affect the DNA repair gene down-regulation. This could be depending on the protein levels, as in the present study, significant reduction in the protein levels in the exposed workers carrying homozygous *Glu* allele compared to the workers carrying the other genotypes. Those workers were also proved to have high levels of BPDE-albumin adducts and tumor biomarkers.

Alpha1-Antitrypsin (A1AT) is a protein produced primarily in the hepatocytes. More than 100 different allelic variants of the A1AT gene have been described. M alleles are the normal variants of the gene. Other common variants are S and Z (Crystal 1989). The present study detected significant increases in the levels of BPDE-albumin adduct and lung tumor biomarkers in the workers with S and Z mutations of theA1AT gene compared to the workers carrying *MM* wild type. The increased frequency of mutant genotype alleles among workers with high BPDE-Alb adduct levels may be attributed to the ability of PAHs metabolites to bind to cellular proteins and DNA, resulting in cell damage and mutations (Ramesh et al. 2010). A previous study on Egyptian asthmatic patients reported that the highest frequency of mutant A1AT allele was found in the patients compared to healthy control (Daabis et al. 2014). Interestingly, our study was the first study that describes the association between A1AT gene mutation and the levels of PAHs adducts in exposed workers.

Exposure to certain PAHs compounds in the general environment is associated with decreases in the lung functions and increases of airway inflammation (Barraza-Villarreal et al. 2014). Statistically significant findings in this study were markedly up-regulation of A1AT mRNA and protein expression among workers with high BPDE-Alb adducts level (≥ 15 ng/ml) compared to those with low BPDE-Alb adducts level workers (< 15 ng/ml). This findings were in consistent with the previous results denoted that, patients with evidence of inflammation had a higher A1AT levels than seen in the absence of inflammation (Sanders et al. 2018; Ferrarotti et al. 2012; Ottaviani et al. 2011). Moreover, the significant positive correlations detected in the present study between BPDE-Alb adducts level and both A1AT protein level and mRNA gene expression among exposed workers, could support that PAHs can damage DNA by producing BPDE adduct, as mentioned by Benhamou et al. (2000). This ensures the high risk of DNA damage and the possibility of increasing the susceptibility to develop cancers in exposed workers to PAHs, unless a refined repair system has evolved to eliminate DNA adducts from the genome.

Since A1AT is characterized by a rapid increase in its concentration during the acute phase of inflammation, to response to protease–anti-protease imbalance, it results in an increase in proteolytic activity in the inflammatory site (Jeppsson and Franzén 1982). In addition, A1AT protects the alveolar tissue from destruction by neutrophil elastase (NE), a powerful protease that destroys the major structural proteins of the cells (Denden et al. 2012). Subsequently, other authors have found that serum A1AT levels are associated with the clinical stage of the disease (Stamatiadis et al. 1990). Moreover, A1AT genotyping and the serum A1AT levels may be useful markers for distinguishing between early and advanced stages of lung cancer in PAHs exposed workers.

Our results showed also a significant positive correlation between PAHs level and both A1AT protein level and mRNA gene expression among exposed workers. As BPDE; a metabolite product of PAHs can damage DNA by producing BPDE adduct (Jeppsson and Franzén 1982). This ensures the risk of DNA damage and development of lung cancer on exposure to PAHs, unless a refined repair system has involved to eliminate DNA adducts from the genome.

## Conclusion

Aluminum workers may be at risk of lung cancer development as a result of exposure to PAHs despite that the air measurement of PAHs were within the permissible limit of exposures. The individual PAHs concentrations were attributed to combustion activities. Exposure to PAHs forms DNA adducts that are associated with increased individual susceptibility to cancer. In addition, aluminum workers are subjected to defect in DNA repair gene due to the inflammation caused by PAHs, exposure. This DNA repair defect will increase the probability of A1AT mutation which in term increases lung cancer susceptibility. Thus, SCCAg, CCNB1 and DNA repair genes estimation can be used as predictor biomarkers for early-staged lung cancer susceptibility in PAHs exposed workers. The current study recommended biological monitoring as it provides more pertinent information than air sampling data.

## Data Availability

The authors confirm that raw date are available from corresponding author upon reasonable request.
